# Supplementary material for: Photonic integrated beam delivery for a rubidium 3D magneto-optical trap
Source: Nat Commun. 2023 May 29;14:3080. doi: 10.1038/s41467-023-38818-6 (PMC10227028; doi:10.1038/s41467-023-38818-6)
Supplement: Supplementary file 1 — Supplementary Information [file 41467_2023_38818_MOESM1_ESM.pdf]

Supplementary Information

Photonic integrated beam delivery for a rubidium 3D magneto-optical trap

Andrei Isichenko[1], Nitesh Chauhan[1], Debapam Bose[1], Jiawei Wang[1], Paul D. Kunz[2], and Daniel J. Blumenthal[1*]

[1] Department of Electrical and Computer Engineering, University of California Santa Barbara, Santa Barbara, CA 93106 USA

[2] DEVCOM U.S. Army Research Laboratory, Adelphi, MD 20783 USA

* Corresponding author (danb@ucsb.edu)

Supplementary Note 1: Introduction

In this Supplementary Information, we discuss further details with respect to the laser cooling beam delivery PIC device fabrication process, including the waveguide, slab expander, and grating emitter design. We also describe more about the PIC optical losses, strategies for loss reduction, beam profiles, and operation and characterization of the MOT.

Supplementary Note 2: Beam delivery device fabrication

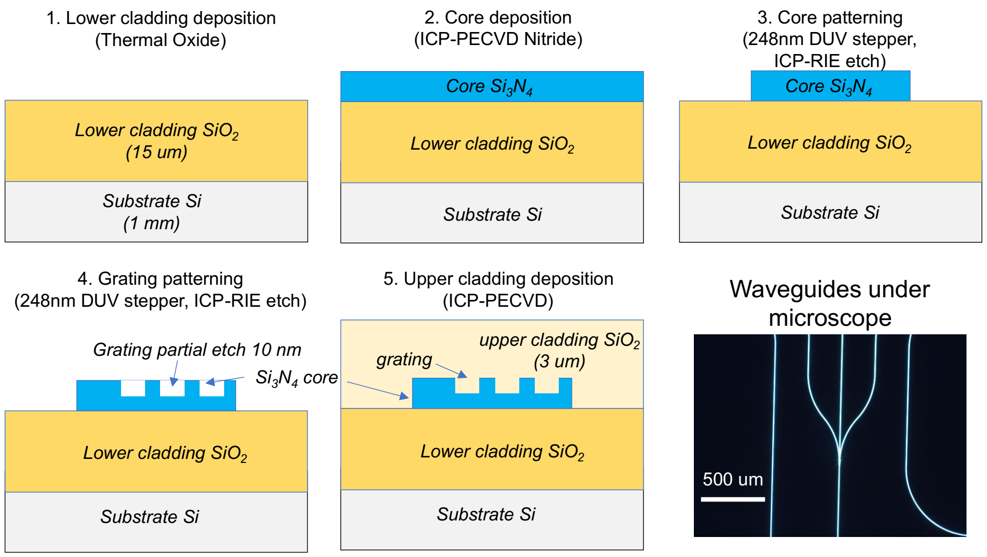

Supplementary Fig. 1. Beam delivery PIC fabrication process flow. Step 1: lower SiO_2 cladding deposition. Step 2: Silicon nitride core deposition. Step 3: Silicon nitride core etch. Step 4: grating layer partial etch. Step 5: Upper oxide cladding deposition. Image of the 1x3 splitter of the fabricated PIC.

Exposure calibration procedure. To calibrate the lithography steps for the waveguide and grating etches, two sets of focus exposure array jobs with the ASML PAS 5500 stepper tool were done using a wafer with silicon nitride on top of thermal oxide on a silicon substrate. The doses of the center elements were 16 mJ cm^{-2} and 25 mJ cm^{-2}, respectively. The focus offset difference between the elements was 0.2 µm for both. An image quality control (IQC) procedure was run on the tool to ensure that the absolute focus exposure correction while running a job would be less than 20 nm. After development of the resist and etching of the antireflective coating layer, the nitride layer was etched with our standard recipes for the waveguide and grating etches[1,2], as shown in Supplementary Fig. 1. Subsequently, the wafer was cleaned and measurements of the waveguide and grating dimensions were taken with a SEM. The elements of the focus exposure array with the best quality were used to inform the conditions for the PIC devices of this work.

Supplementary Note 3: Beam delivery device design

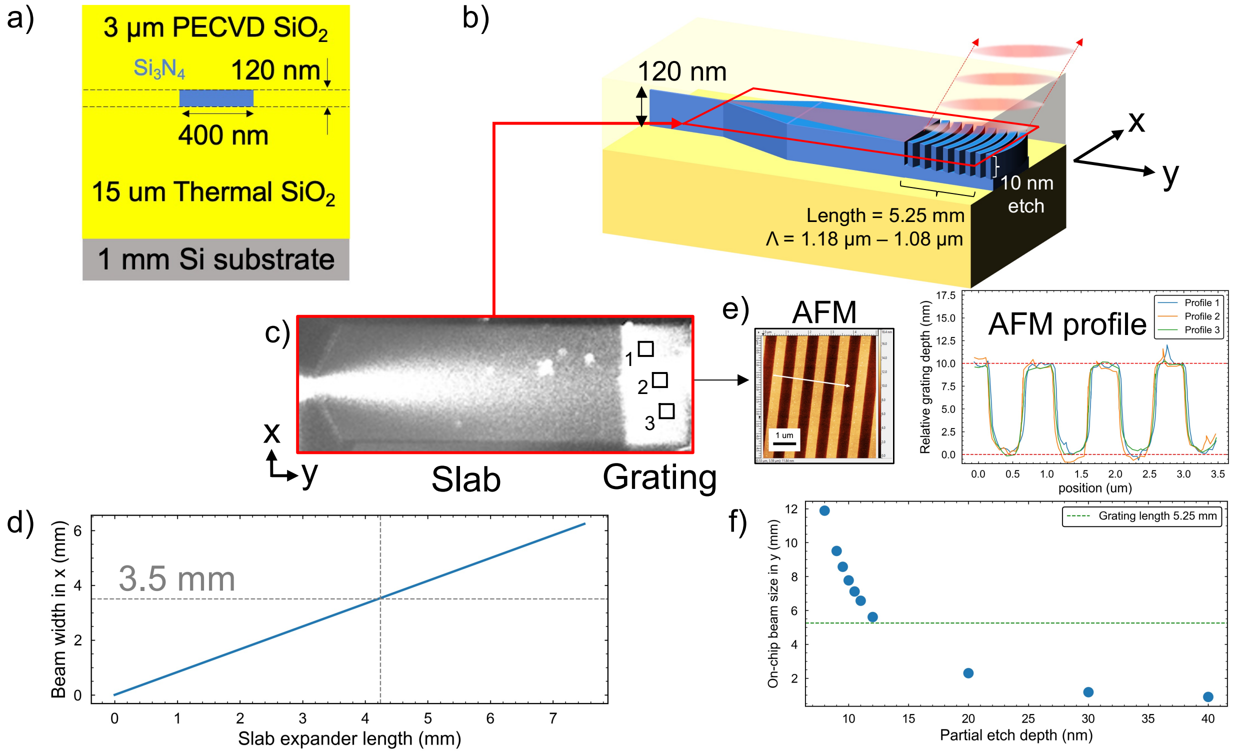

Supplementary Fig. 2. Beam delivery device design. a) Waveguide dimension for single-mode operation at 780 nm. b) Slab expander and grating emitter. c) Image of the slab expander grating emitter illuminated with 780 nm light. d) Simulation of the slab expander rate of beam expansion. The expander enables reaching an on-chip beam width of 3.5 mm after 4.2 mm of expansion. e) AFM images at three profile locations shows that the grating emitter partial etch depth is relatively uniform across the emitter. f) Simulation of achievable on-chip beam size in the y dimension for different grating partial etch depths.

Grating design and modeling. The laser cooling beams are formed using three large-area, chip-to-free-space grating couplers. These couplers are formed in a material system composed of a silicon nitride device layer and two silicon dioxide cladding layers. Historically, grating couplers have

been used in integrated photonics for a relatively small subset of tasks, the most well-known of which is likely the coupling of light between chip-scale waveguides and optical fibers[3]. Grating couplers are ideal for interfacing between planar devices and their surrounding environment. Due to the careful control over the grating's k-vector, the angle at which light is either emitted or transmitted can be targeted with a high degree of precision. When light propagating within a chip diffracts from a grating written into its path, the angle at which the light diffracts relative to the chip's surface normal may be calculated analytically. If we assume that the light is initially propagating within the x-y plane, and that the z-axis represents the direction normal to the chip's surface, then the diffraction angle is given as:

$$\theta = \tan^{-1}\left(\frac{\sqrt{{k_{\mathrm{x,o}}}^2 + {k_{\mathrm{y,o}}}^2}}{k_{\mathrm{z,o}}}\right) \tag{1}$$

where

$$\begin{aligned} k_{\mathrm{x,o}} &= k_{\mathrm{x}} - K_{\mathrm{x}} \\ k_{\mathrm{y,o}} &= k_{\mathrm{y}} - K_{\mathrm{y}} \\ k_{\mathrm{z,o}} &= k_{\mathrm{z}} - K_{\mathrm{z}} \end{aligned} \tag{2}$$

and $k_{\mathrm{z,o}} = \sqrt{(2\pi/\lambda)^2 - {k_{\mathrm{x,o}}}^2 - {k_{\mathrm{y,o}}}^2}$. In these expressions, k_{x} and k_{y} are the x- and y-components of the initial k-vector of the light, and K_{x} and K_{y} are the x- and y-components of the grating's k-vector, respectively. Additionally, $k_{\mathrm{x,o}}$, $k_{\mathrm{y,o}}$, and $k_{\mathrm{z,o}}$ represent the three components of the diffracted light's k-vector in free-space. For the MOT, the three beams diffracted from the chip are required to each be orthogonal to one another. This requires that for each grating the diffraction angle relative to the surface normal equals 54.7° and the slabs spaced 120° apart from each other. The center of each grating emitter is placed on 13.5 mm circle to ensure the 9.45 mm intersection spot which enables enough clearance for waveplates and cell wall.

At the interface between the waveguide, which provides two-dimensional confinement, and the slab expander, which only provides confinement along one axis, the optical wave begins to diverge along the direction which is both transverse to its propagation and parallel to the chip's surface normal. This divergence can be represented mathematically as a spatially dependent distribution of optical k-vectors, given as:

$$\begin{aligned} k_{\mathrm{x}}(x,y) &= k_{\mathrm{o}} \cos(y/x) \\ k_{\mathrm{y}}(x,y) &= k_{\mathrm{o}} \sin(y/x) \end{aligned} \tag{3}$$

In this expression, x and y are the coordinates within the nitride slab relative to the point at which the waveguide-to-slab transition occurs. This means that because the light is required to be emitted in a specific direction regardless of position, the grating coupler's k-vector must vary spatially to compensate for the varying components of the optical k-vector. This requirement may be represented mathematically as:

$$K_{\mathrm{y}} = k_{\mathrm{y}} = k_{\mathrm{o}} \sin(\tan^{-1}(y/x))$$
$$K_{y} = k_{\mathrm{y}} - k_{\mathrm{o}} \sin(y/x) \quad (4)$$
$$K_{\mathrm{x}} = k_{\mathrm{x}} - \frac{k_{\mathrm{o}} \tan(54.7°)}{\sqrt{1 + \tan(54.7°)}}$$

After these local maps of grating period and direction have been calculated, the spatially variant grating may be generated numerically by beginning a single grating line at a chosen starting point and "walking forward", iteratively defining its movement and width. This process may be repeated for an arbitrary number of grating lines until the chosen grating area has been defined. We thus generate curved gratings which take slab mode and produce a flat intensity profile in free space. Based on results from a previous fabrication run we re-used a photolithography mask with a grating length of 5.25 mm (corresponding to a cross-sectional beam width of 2.5 mm). The grating etch depth required for an on-chip beam width of 5.25 mm was simulated to be 12 nm in a 120 nm core. A 10 nm grating etch depth was chosen considering a potential 20% etch depth variation. Subsequent measurements of AFM show that the etch depth variation is smaller than expected (Supplementary Fig. 2(d)). The grating coupling strength is lowest at the grating input and progressively increases as the light propagates in the grating region. The grating period and duty cycle are designed to progress from 1.18 μm – 1.08 μm and 10% - 50% from front to end of the grating respectively (Supplementary Fig. 2(b)).

Supplementary Note 4: Beam delivery device characterization and future improvements

Optical losses in the beam delivery. We measured the optical losses at each major component on another beam delivery PIC device of the same fabrication run (see Supplementary Table 1). The individual losses of facet, MMI, slab expander and grating were extracted by iteratively measuring and dicing the device in a cutback method. First, the loss of full-size device was measured. Next, the grating was diced from slab and an integrating sphere was used to measure the slab output and extract the grating loss. Finally, waveguide output without the slab was measured to extract the facet coupling loss. The propagation loss was measured by subtracting the facet loss from a long loop around waveguide. For the single grating emitter measured, the total loss from the waveguide input to the grating output beam was 12.5 dB. The MMI splitter excess loss was inferred from the performance of one of the beams of the PIC device used in this work.

The rightmost column of Supplementary Table 1 estimates the achievable losses in future beam delivery PIC designs based on simulations, test structure measurements, and values from literature. The MMI splitter excess losses originate from fabrication imperfections due to the small dimensions of MMI which in the future we will address by replacing the MMI with an evanescent coupler which we have measured to have <0.3 dB loss. The fiber-to-chip coupling loss can be reduced by using tapered mode matchers, facet polishing, or other mode-matching structures[4]. Next, our waveguide is designed to support a TE mode with an estimated loss of ~0.3 dB cm^{-1}. By increasing the waveguide width we can operate in TM mode which has been shown to achieve a lower loss[5]. We estimate that with a loss of 0.15 dB cm^{-1} we can achieve a total propagation loss of 0.7 dB. Lastly, the grating emitter output power is distributed over different diffracted orders which affects the overall beam delivery efficiency (Supplementary Figure 3(b)). We define the power in the target diffracted order as P_0 and the power in the other diffracted order (near vertical)

as P_{+1} and a ratio $R = P_{+1}/P_0$. From simulation we find that we can improve the diffraction efficiency into the 0 order and reduce R by adjusting the grating partial etch depth. We find that at an optimal etch depth we can reach $R = 0.5$ (supplementary Figure 3(c)). Assuming that the fraction of total optical power delivered by the emitter is unchanged, we estimate that we can reduce the grating output loss by 1.1 dB. This requires accurate control of the etch depth and with proper calibration a tolerance of <0.5 nm can be achieved. Furthermore, our simulation predicts that for a 10 nm etch depth and a 5.25 mm length grating section, 20% of the power is not diffracted and scatters. By extending the grating length to 7 mm we predict that we can increase the diffracted power and reduce the total grating output loss by 1.7 dB. We estimate a total achievable beam deliver loss (input fiber to all three beams) of 7.2 dB. Further improvements may be possible by using a bottom reflector below the grating[6] and using grating inverse design[7].

Supplementary Table 1. Beam delivery loss budget.

Location on PIC	Measured Loss	Estimated Achievable Loss
Fiber to input waveguide	3.8 dB	0.3 dB[4]
1x3 splitter excess loss and waveguide propagation loss	3.3 dB	1.0 dB
Waveguide to slab expander	0.7 dB	0.7 dB
Slab to free-space grating output	8.0 dB	5.2 dB
Total beam delivery loss input fiber to all PIC beams	15.8 dB	7.2 dB

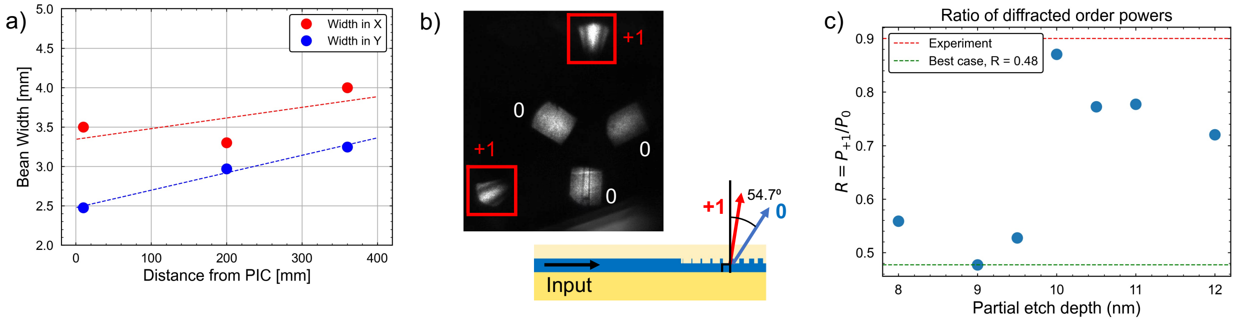

Supplementary Fig. 3. Grating emitter beam characterization. a) Measurements of the beam collimation for PIC beam 1 (B1). The beam has divergence angles of 0.16° and 0.35° in the x- and y-dimensions as defined in Figure 2(d) of the main text. The corresponding effective Rayleigh lengths are 125 and 113 cm in the x- and y- axes, respectively. b) Image of the higher-order mode for beams 2 and 3 (B2, B3) with imaging paper sheet above the beam intersection point. The +1 order for the third beam (not shown) has a similar profile. c) Simulation for ratio of power of diffracted orders for varying partial etch depths.

Beam collimation and intensity profile. The beams profile and angle of the intersecting beams was measured with a camera that imaged a translucent paper sheet held above the PIC surface at

varying distances. The measured beam width (defined as the e^{-2} diameter) for beam B1 as a function of the distance from the emitter is shown in Supplementary Fig. 3 (a). We note that beam B3 has a non-uniformity in its intensity profile which we attribute to imperfect photolithography exposure which effectively reduced the width of the waveguide at the input to the slab expander. We attribute the fact that the MOT cloud is not centered inside the beam overlap volume to this beam non-uniformity.

Supplementary Note 5: MOT system and characterization

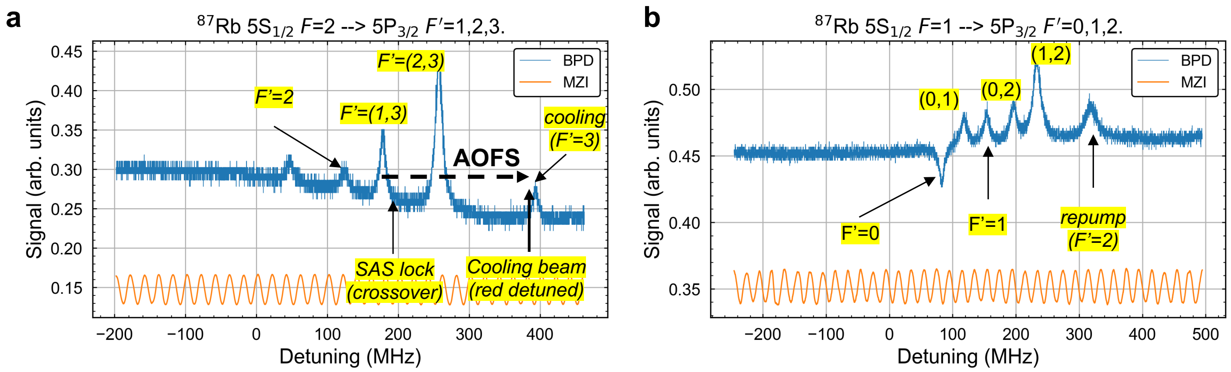

Supplementary Fig. 4. Laser stabilization for MOT demonstration. a) Frequency sweep of the cooling laser across the ^{87}Rb $5S_{1/2}\ F = 2 \rightarrow 5P_{3/2}\ F' = 1,\ 2,\ 3$ transitions. An acousto-optical frequency shifter (AOFS) is used to shift the laser locked to the $F' = (1,3)$ peak to achieve a red-detuning with respect to the $F' = 3$ cooling transition. An unbalanced Mach-Zehnder interferometer (MZI) with FSR 20 MHz is used to calibrate the laser frequency sweep while the saturation absorption spectroscopy signal is recorded on a balanced photodetector (BPD). b) Frequency sweep across the ^{87}Rb $5S_{1/2}\ F = 1 \rightarrow 5P_{3/2}\ F' = 0,\ 1,\ 2$ transitions. The repump laser is aligned to the $F' = 2$ cooling transition.

MOT laser system. A frequency sweep of the relevant ^{87}Rb D_2 line hyperfine transitions of the saturation absorption spectroscopy setup is shown in Supplementary Fig. 4. The cooling laser is frequency stabilized to a cross-over peak (left panel) and the acousto-optical frequency shifter (AOFS) is used to control the ~200 MHz shift for detuning adjustment. The repump laser is free-running and aligned with respect to the repump transition by maximizing the signal of the MOT fluorescence.

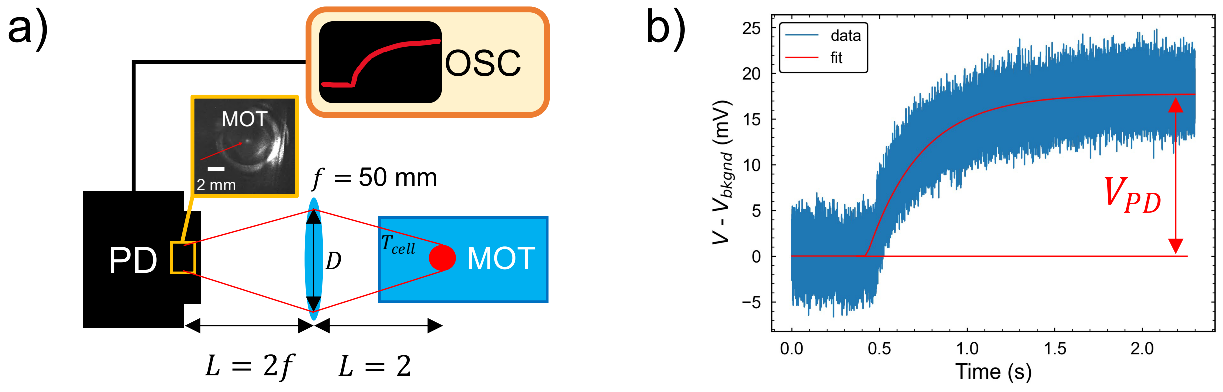

Supplementary Fig. 5. Atom number fluorescence measurement. a) Schematic of the fluorescence collection onto a photodetector (PD). b) PD loading curve trace with scattered light background subtracted. The magnetic field is enabled at $t = 0.4$ seconds and the loading time constant from the fit is $\tau = 307$ ms.

MOT fluorescence measurements. We measure the MOT atom number and loading rate by measuring the atom cloud fluorescence with a photodetector. The number of atoms is determined by the optical power of the atom cloud fluorescence P_{in} emitted at the wavelength 780 nm over a solid angle Ω. The atom number from fluorescence detection is given by

$$N = \frac{4\,\pi\,P_{\text{in}}}{\Omega \frac{hc}{\lambda}\,R_{\text{scat}}} \tag{5}$$

where the photon scattering rate is given by[8]

$$R_{\text{scat}} = \left(\frac{\Gamma}{2}\right) \frac{\frac{I_{\text{tot}}}{I_{\text{sat}}}}{1 + \frac{I_{\text{tot}}}{I_{\text{sat}}} + 4\,\left(\frac{\Delta}{\Gamma}\right)^2} \tag{6}$$

where the detuning $\Delta = 2\pi\,\delta f_{\text{laser}}$ and δf_{laser} is the frequency detuning in MHz of the cooling laser with respect to the ^{87}Rb $5S_{1/2}\ F = 2 \rightarrow 5P_{3/2}\ F' = 3$ transition. In the high intensity limit $I_{\text{tot}} \gg I_{\text{sat}}$, $R_{\text{scat}} = 0.5\,\Gamma = (2\tau)^{-1}$ where τ is the lifetime of the excited state. In this regime, on average each atom completes an absorption and emission cycle during 2τ time scale[9]. Given a scattering rate R_{scat}, atom number N, and photon energy hc/λ, a MOT cloud will fluoresce at a power given by

$$P = N\,R_{\text{scat}} \frac{hc}{\lambda} \tag{7}$$

By using a focusing lens to collect a portion of the fluorescence power P_{in} onto a photodetector of responsivity $R(\lambda)$ and transimpedance gain G, the output photodetector voltage is

$$V_{\text{PD}} = P_{\text{in}} R(\lambda) G \eta \tag{8}$$

Where η is the efficiency of the light collection which includes the transmission through surfaces of the focusing lens and the glass cell. The fraction of the fluorescence collected is determined by the solid angle set by the lens, assuming that the fluorescence is uniformly radiated over a solid angle of 4π. For a lens of clear aperture diameter D a distance L away from the cloud, the solid angle is given by[9]

$$\Omega = \int_0^{2\pi} \int_0^{\theta_F = \arctan\left(\frac{D}{2L}\right)} \sin(\theta)\, d\theta\, d\phi = 4\pi \sin^2\left(\frac{1}{2}\arctan\left(\frac{D}{2L}\right)\right) \tag{9}$$

When the MOT is switched on (by enabling the magnetic field), the number of atoms will increase according to

$$N(t) = N_0\left(1 - e^{-t/\tau_{\text{load}}}\right) \tag{10}$$

where τ_{load} is the atom loading time in the trap and N_0 is the steady-state (i.e. maximum) atom number in the MOT. Given a trace of the photodiode voltage during a loading rate measurement, the maximum atom number can be measured:

$$N_0 = \frac{V_0}{\eta R(\lambda) G} \frac{1}{R_{\text{scat}} hc/\lambda} \frac{4\pi}{\Omega} \tag{11}$$

For a measured difference in $V_{\text{PD}} = V_{\text{signal}} - V_{\text{bkgnd}}$ =17.7 mV, the collected fluorescence power is 8.0 ± 0.7 nW. The error from the detector noise equivalent power (NEP) is 2.1pW Hz$^{-1/2}$ which corresponds to an RMS noise of 0.7 nW for a bandwidth of 100 kHz. This corresponds to a peak atom number $N_0 = 1.3 \pm 0.2$ million atoms for an input power to the PIC of 180 mW and a detuning of $\Delta = -2.4\ \Gamma$. Supplementary Table 2 contains parameters used in the calculation of the atom number. The fluorescence measurement schematic is shown in Supplementary Figure 5(a). The fluorescence is collected with a lens (f = 50 mm) and a switchable gain Si photodetector (Thorlabs PDA36A). The detector is calibrated at the gain setting used (70 dB) and measured to have a linear response for the range of collected fluorescence power. For the voltage trace in Supplementary Figure 5(b), the trapping and repump light are kept on and the magnetic field trap is enabled at $t = 0.4$ seconds. Turning on the magnetic field has a negligible effect on the collected fluorescence signal.

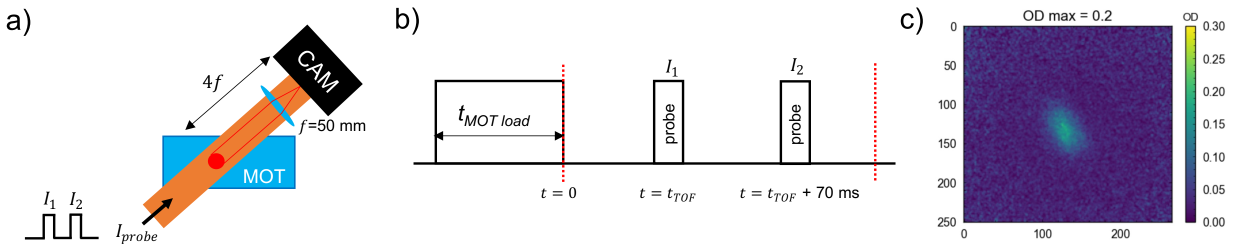

Supplementary Fig. 6. MOT absorption imaging. a) Schematic of the absorption imaging setup. Dimensions not to scale. b) Timing sequence for the MOT. c) OD image for $t_{\mathrm{TOF}} = 0.1$ ms with axes corresponding to camera pixels.

Absorption imaging. We use absorption imaging to measure the temperature of the cloud and the atom number. The MOT cloud is illuminated with probe beam light resonant ($\Delta_{\mathrm{probe}} \approx 0$) with the $F = 2 \rightarrow F' = 3$ transition (Supplementary Figure 6(a)). Absorption imaging requires two sequential images, I_1 with atoms and I_2 without atoms. I_1 is the image of the probe beam flashed (duration 0.5 ms) at a time t_{TOF} after the MOT cooling/repump beam and magnetic field shut-off. I_2 is recorded at $t_{\mathrm{TOF}} + 70$ ms when the MOT has dissipated. The cooling and repump beams are shuttered with the 780 nm semiconductor optical amplifier (SOA) and the probe is shuttered with an AOFS. As the probe beam passes through the cloud, the intensity I_2 of the beam will be attenuated according to Beer's law:

$$I_1 = I_2 e^{-\sigma \int n(x,y,z) dx} \tag{12}$$

Where $n(x, y, z)$ is the atom number density which can be integrated along the beam axis x to obtain the integrated column density and σ is the scattering cross section of a two-level atom given by

$$\sigma = \sigma_0 \frac{1}{1 + \frac{I_{\mathrm{probe}}}{I_{\mathrm{sat}}} + 4\left(\Delta_{\mathrm{probe}}/\Gamma\right)^2} \tag{13}$$

Supplementary Table 1 contains parameters used in the calculation and experiment. The optical density of the cloud is defined such that

$$I_1 = I_2 e^{-\mathrm{OD}} \Rightarrow \mathrm{OD} = -\ln\left(\frac{I_1}{I_2}\right) = \sigma \int n(x, y, z) dx \tag{14}$$

The atom number density $n(x, y, z)$ follows a three-dimensional Gaussian distribution

$$n(x, y, z) = n_0 \exp\left(-\frac{x^2}{2\sigma_{\mathrm{x}}^2}\right) \exp\left(-\frac{y^2}{2\sigma_{\mathrm{y}}^2}\right) \exp\left(-\frac{z^2}{2\sigma_{\mathrm{z}}^2}\right) \tag{15}$$

where $\sigma_{\mathrm{x}}, \sigma_{\mathrm{y}}, \sigma_{\mathrm{z}}$ are the root-mean-square widths of the cloud and n_0 is the peak atom cloud density. We estimate the size of the third dimension (along the imaging axis x) from the geometric

mean of the sizes of the other two dimensions ($\sigma_{\mathrm{x}} = \sqrt{\sigma_{\mathrm{y}}\sigma_{\mathrm{z}}}$)[10]. The measured OD image can be modeled as

$$\mathrm{OD}(y,z) = \mathrm{OD}_{\mathrm{max}} \exp\left(-\frac{x^2}{2\sigma_{\mathrm{x}}^2}\right) \exp\left(-\frac{y^2}{2\sigma_{\mathrm{y}}^2}\right) \tag{16}$$

where $\mathrm{OD}_{\mathrm{max}} = \sigma\, n_0 \sqrt{2\pi\sigma_{\mathrm{y}}\sigma_{\mathrm{z}}}$ is the peak value of the OD image extracted from the camera which can be used to extract n_0. The cloud widths are extracted by summing over the raw OD image along each axis and fitting to a one-dimensional Gaussian. The widths in camera pixels are converted to meters based on the measured magnification of the imaging system during which the camera was focused on the MOT cloud fluorescence in steady-state MOT operation. The procedure is repeated for several different time delays between the MOT shut-off and the start of the I_1 pulse corresponding to different values of time-of-flight time (t_{TOF}). The temperature of the cloud along each axis $T_{\mathrm{y}}, T_{\mathrm{z}}$ can be determined from the ballistic expansion by plotting the cloud width as a function of t_{TOF},

$$\sigma_{\mathrm{y}}^2 = \sigma_{\mathrm{y,i}}^2 + \frac{k_{\mathrm{B}}T_{\mathrm{y}}}{m} t_{\mathrm{TOF}}^2 \tag{17}$$

$$\sigma_{\mathrm{z}}^2 = \sigma_{\mathrm{z,i}}^2 + \frac{k_{\mathrm{B}}T_{\mathrm{z}}}{m} t_{\mathrm{TOF}}^2 \tag{18}$$

where $\sigma_{\mathrm{y,i}}$ and $\sigma_{\mathrm{z,i}}$ are the initial widths of the cloud, k_{B} is the Boltzmann constant, and m is the mass of a single ^{87}Rb atom. The total number of atoms is the volume integral of the atom density

$$N = \int\int\int n(x,y,z)\, dx\, dy\, dz = \int\int \frac{\mathrm{OD}(y,z)}{\sigma}\, dy\, dz = \frac{\mathrm{OD}_{\mathrm{max}}}{\sigma}\, 2\pi\sigma_{\mathrm{y}}\sigma_{\mathrm{z}}. \tag{19}$$

For images taken at $t_{\mathrm{TOF}} = 0.1$ ms, $\mathrm{OD}_{\mathrm{max}} = 0.2$. We measure $N = 0.13 \pm 0.05$ million atoms for a power into the PIC of 65 mW. Note that this power is limited by the total output power of the SOA and therefore explains the lower atom number compared to the peak atom number measured with fluorescence detection. In these calculations the raw OD image from the camera is subject to background and scattered probe light. Any light that is collected by the camera that cannot be absorbed by the cloud will reduce the measured OD. The images are recorded with the Thorlabs DCC3260M CMOS monochrome camera operated with an external voltage trigger.

Supplementary Table 2. Parameters for fluorescence and absorption imaging measurements.

Parameter	Meaning	Value	Ref
λ	^{87}Rb D_2 transition wavelength	780.241 nm	8
Γ	^{87}Rb natural linewidth for the D_2 ($5^2S_{1/2} \rightarrow 5^2P_{3/2}$) transition	$2\,\pi$ (6.066 MHz)	8
σ_0	^{87}Rb resonant cross section intensity for the $F = 2 \rightarrow F' = 3$ and isotropic light polarization.	$1.36 * 10^{-9}$ cm^2	8
I_{sat}	^{87}Rb saturation intensity for the $F = 2 \rightarrow F' = 3$ and isotropic light polarization.	3.577 mW cm^{-2}	8
Δ	(Angular) frequency detuning from the $F = 2 \rightarrow F' = 3$ transition	-2π (14.6 ± 1.0 MHz)	
P_{avg}	Average power of each PIC beam	1.00 ± 0.01 mW	
A_{beam}	Area of each PIC beam, using the $1/e^2$ beam width for each beam axis and assuming a rectangle.	0.09 cm^2	
I_{beam}	Average intensity of each PIC beam before the glass cell	$\frac{P_{\text{avg}}}{A_{\text{beam}}}$	
T_{cell}	Measured transmission through a single face of the uncoated glass vacuum cell.	0.9	
I_{tot}	Sum of the intensities of the six trapping beams at the location of atoms. Three 3 input beams and 3 retroreflected (RR) beams. Assume RR reflection nearly 1.	$3\,T_{\text{cell}}\,I_{\text{beam}} + 3T_{\text{cell}}^2 I_{\text{beam}}$	
Ω	Solid angle of collected light. Distance from the $f = 50$ mm lens to MOT is $L = 100 \pm 10$ mm. The lens clear aperture is $D = 22.9 \pm 0.5$ mm.	0.04 ± 0.01 sr	
η	Light collection efficiency, assuming the transmission through the B-coated lens is nearly 1.	$\eta = T_{\text{cell}} = 0.9$	
$R(\lambda)$	Responsivity of the fluorescence photodetector	0.48 A W^{-1}	11
NEP	Photodetector noise equivalent power (NEP) at the gain setting (70 dB) used during the measurement	2.1 pW Hz$^{-1/2}$	11
G	Transimpedance gain of the fluorescence photodetector. The measured calibrated value of the gain is within 5% of the specification.	$4.75 * 10^6$ V A^{-1}	11
Δ_{probe}	(Angular) frequency detuning of the probe beam from the $F = 2 \rightarrow F' = 3$ transition during absorption imaging measurements	2π (0 ± 2 MHz)	
I_{probe}	Measured probe beam intensity for absorption imaging measurements	$(0.1\ \pm 0.01)\,I_{\text{sat}}$	
M	Measured magnification in the absorption imaging system	0.9 ± 0.1	

Comparison of atom number measurements. During absorption imaging TOF measurements the booster optical amplifier (BOA) was used to shutter the optical power input to the beam delivery PIC which outputs a maximum power of 65 mW into the PIC corresponding to an average power per PIC beam of 0.460 mW. We did not optimize for the atom number during the absorption imaging measurements. During the fluorescence measurements a tapered amplifier (TA) was used, allowing for reaching over 180 mW into the PIC. We measured the fluorescence atom number for several input powers into the PIC, corresponding to $I_{\mathrm{tot}}/I_{\mathrm{sat}} \in (6, 20)$ shown in Supplementary Figure 7. At input powers into the PIC similar to that of absorption imaging setup, we measure $N = 0.5 \pm 0.1$ million atoms. Furthermore, during the absorption imaging the atom number was not continuously optimized. We expect that repump frequency drift, repump power, and cooling laser locking was not optimal. We observe a saturation of the MOT fluorescence power at a repump intensity above 12 mW cm^{-2} while we can only reach 3 mW cm^{-2} for peak atom number measurements and 1.5 mW cm^{-2} for absorption imaging measurements. As the purpose of this work is to demonstrate the PIC-based delivery of all the beams for MOT operation, the limited repump intensity likely limited the achievable atom number for both measurements. In the future we will use a sideband modulator to generate the repump from the cooling laser by applying ~6.8 GHz sidebands.

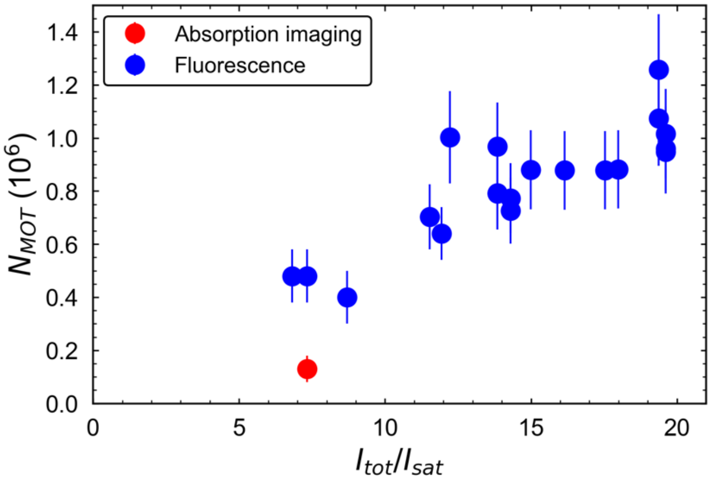

Supplementary Fig. 7. Atom number for different cooling light intensities. Atom number as a function of cooling light intensity measured with different methods for different powers into the PIC. Error bars are the standard deviation from error propagation of uncertainties in Supplementary Table 1.

Supplementary Note 6: Comparison of integrated beam delivery methods for 3D MOT cold atom systems.

Atom number and trapping beam overlap volume. The number of atoms trapped in a MOT depends on the overlap volume of the cooling beams. This is because a larger beam volume in each dimension of the trap is related to the stopping distance available to cool the atoms and therefore the maximum speed of the atoms that can be part of the MOT. The beam overlap volume of our PIC-based beam delivery approach is 22 mm^3. For our trap volume calculation we extract the approximately rectangular contour of the intensity profile of beam B1 at which the intensity falls to $I = I_{\mathrm{sat}}$ similar to the beam diameter definitions from previous work[12]. This rectangle has a

shape $w_{p1} = 3.45$ mm by $w_{p2} = 2.50$ mm. We calculate the beam overlap volume by assuming a rectangular prism with volume $V = w_{p1}w_{p2}^2 = 22$ mm^3. This volume is ~2 times smaller than the volume used for a comparable atom number GMOT as shown in Supplementary Figure 8. Furthermore, these GMOTs utilize an input free-space beam of diameter 20 mm which requires beam expansion and collimation optics. The shaded region denotes the transition region where previous work[12] has identified a change in the scaling of atom number and trapping volume.

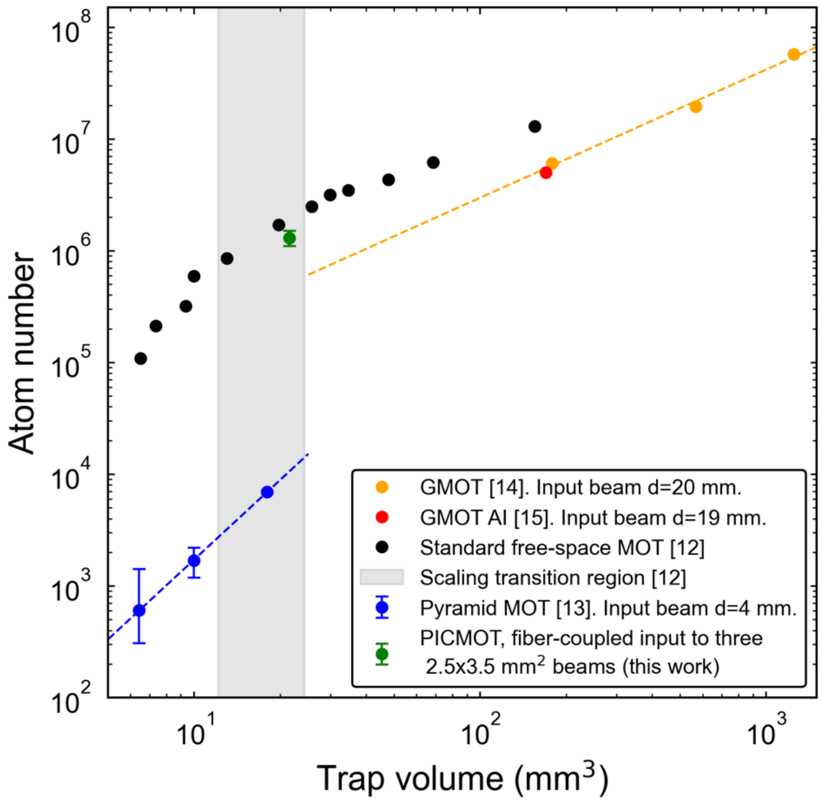

Supplementary Fig. 8. Atom numbers and cooling beam trapping volume for different integrated beam delivery methods. Blue: microfabricated pyramid chip MOTs (pyramid MOT) with error bars from literature[13]. Orange: Diffraction grating MOT (GMOT) with an approximate scaling law[14]. Red: diffraction grating MOT used in a cold atom interferometer sensor[15]. Green: photonic-integrated-circuit MOT (PICMOT) of this work where the error bar is the standard deviation. Black: standard 6-beam free-space MOT in which the atom number vs. trapping beam diameter d was varied[12], using trap volume $V = d^3$ and error bars omitted for clarity. Shaded region: transition region between observed scaling laws[12] for atom number and trap volume.

System integration. Diffraction grating MOTs (GMOTs) have proven to be successful approach in the miniaturization of cold atom systems[15]. However, this technology has limitations in the ability to bring cold atom technology to modular, controllable, and scalable systems. A PIC-based platform offers several key advantages. The Si_3N_4 beam delivery chip is compatible with chip-scale laser integration[16]. Directly butt-coupling an edge-emitting chip-scale laser to the beam delivery PIC obviates the need for fiber-to-chip coupling. This could improve the total efficiency of the cold atom device, which can be defined by comparing the electrical power consumption of the integrated laser to the MOT optical trapping intensity and the atom number. It is possible to

extend the system to multiple integrated laser sources and beam emitters as the PIC can be made to support multiple different wavelengths[17,18]. This can be designed to operate in a single silicon nitride thickness (for example 120 nm used in this work) with mask-level changes in the lithography mask.

The PICMOT offers a different configuration for optical access in a chip-scale cold atom system. A comparison of optical access is given in Table 1 of the main text. We consider an atomic vacuum cell in the shape of a rectangular prism and assume that one face of the cell is dedicated to the atomic source and vacuum pump. The four-beam tetrahedral beam configuration of a GMOT places constraints on the optical access for probing and imaging. There are three faces of optical access and the input vertical beam in a GMOT makes it difficult to image a MOT at a non-orthogonal angle[19]. While this can be overcome by machining a central hole in grating chip[20] (hence a fourth face of access), this extends the size of the package that is already limited by the beam expansion optics required for the input beam. On the other hand, the PICMOT allows for four faces of optical access, with an optional fifth face is we consider probe beams emitted by the PIC. This extension of PIC to accommodate other beams, such as probe beams for Raman and two-photon transitions, can be achieved by modifying the lithography mask as described above. Improvements to optical access can also enable collection of a stronger atomic fluorescence signal which may eliminating the need for higher SWaP detectors or complicated imaging schemes[19].

Supplementary References

1. Gundavarapu, S. *et al.* Sub-hertz fundamental linewidth photonic integrated Brillouin laser. *Nat. Photonics* **13**, 60–67 (2019).

2. Chauhan, N. *et al.* Photonic Integrated Si3N4 Ultra-Large-Area Grating Waveguide MOT Interface for 3D Atomic Clock Laser Cooling. in STu4O.3 (Optical Society of America, 2019). doi:10.1364/CLEO_SI.2019.STu4O.3.

3. Taillaert, D. *et al.* Grating Couplers for Coupling between Optical Fibers and Nanophotonic Waveguides. *Jpn. J. Appl. Phys.* **45**, 6071 (2006).

4. Puckett, M. W. & Krueger, N. A. Broadband, ultrahigh efficiency fiber-to-chip coupling via multilayer nanophotonics. *Appl. Opt.* **60**, 4340 (2021).

5. Liu, K. *et al.* Ultralow 0.034 dB/m loss wafer-scale integrated photonics realizing 720 million Q and 380 μW threshold Brillouin lasing. *Opt. Lett.* **47**, 1855–1858 (2022).

6. Zou, J. *et al.* Ultra efficient silicon nitride grating coupler with bottom grating reflector. *Opt. Express* **23**, 26305–26312 (2015).

7. Spektor, G. *et al.* Universal visible emitters in nanoscale integrated photonics. Preprint at http://arxiv.org/abs/2206.11966 (2022).

8. Steck, D. A. Rubidium 87 D Line Data. http://steck.us/alkalidata (2021).

9. Rushton, J. A novel magneto optical trap for integrated atom chips. (University of Southampton, 2015).

10. Mickelson, P. G. Trapping and Evaporation of ^{87}Sr and ^{88}Sr Mixtures. (Rice University, 2010) https://ultracold.rice.edu/publications/MickelsonThesis.pdf.

11. PDA36A(-EC) Si Switchable Gain Detector: User Guide. *Thorlabs* https://www.thorlabs.com/drawings/51dccca82060b18d-D3B9F20D-CB5D-16F3-7FCA795FB41AFBB9/PDA36A-Manual.pdf (2017).

12. Hoth, G. W., Donley, E. A. & Kitching, J. Atom number in magneto-optic traps with millimeter scale laser beams. *Opt. Lett.* **38**, 661 (2013).

13. Pollock, S., Cotter, J. P., Laliotis, A., Ramirez-Martinez, F. & Hinds, E. A. Characteristics of integrated magneto-optical traps for atom chips. *New J. Phys.* **13**, 043029 (2011).

14. Nshii, C. C. *et al.* A surface-patterned chip as a strong source of ultracold atoms for quantum technologies. *Nat. Nanotechnol.* **8**, 321–324 (2013).

15. Lee, J. *et al.* A compact cold-atom interferometer with a high data-rate grating magneto-optical trap and a photonic-integrated-circuit-compatible laser system. *Nat. Commun.* **13**, 5131 (2022).

16. Fan, Y. *et al.* Hybrid integrated InP-Si_3N_4 diode laser with a 40-Hz intrinsic linewidth. *Opt. Express* **28**, 21713–21728 (2020).

17. Chauhan, N. *et al.* Visible light photonic integrated Brillouin laser. *Nat. Commun.* **12**, 4685 (2021).

18. Chauhan, N. *et al.* Ultra-low loss visible light waveguides for integrated atomic, molecular, and quantum photonics. *Opt. Express* **30**, 6960–6969 (2022).

19. McGilligan, J. P. *et al.* Laser cooling in a chip-scale platform. *Appl. Phys. Lett.* **117**, 054001 (2020).

20. Bregazzi, A. *et al.* A simple imaging solution for chip-scale laser cooling. *Appl. Phys. Lett.* **119**, 184002 (2021).
