# Supplementary material for: Photonic integrated beam delivery for a rubidium 3D magneto-optical trap
Source: Nat Commun. 2023 May 29;14:3080. doi: 10.1038/s41467-023-38818-6 (PMC10227028; doi:10.1038/s41467-023-38818-6)
Supplement: Supplementary file 2 — Description of Additional Supplementary Files [file 41467_2023_38818_MOESM2_ESM.pdf]

File name: Supplementary Movie 1

Description: PICMOT demonstration while (timestamp 0:00) manipulating the ratio of currents of the magnetic field coils for magnetic field shimming and (timestamp 0:09) changing the polarization state of the light into the PIC.
